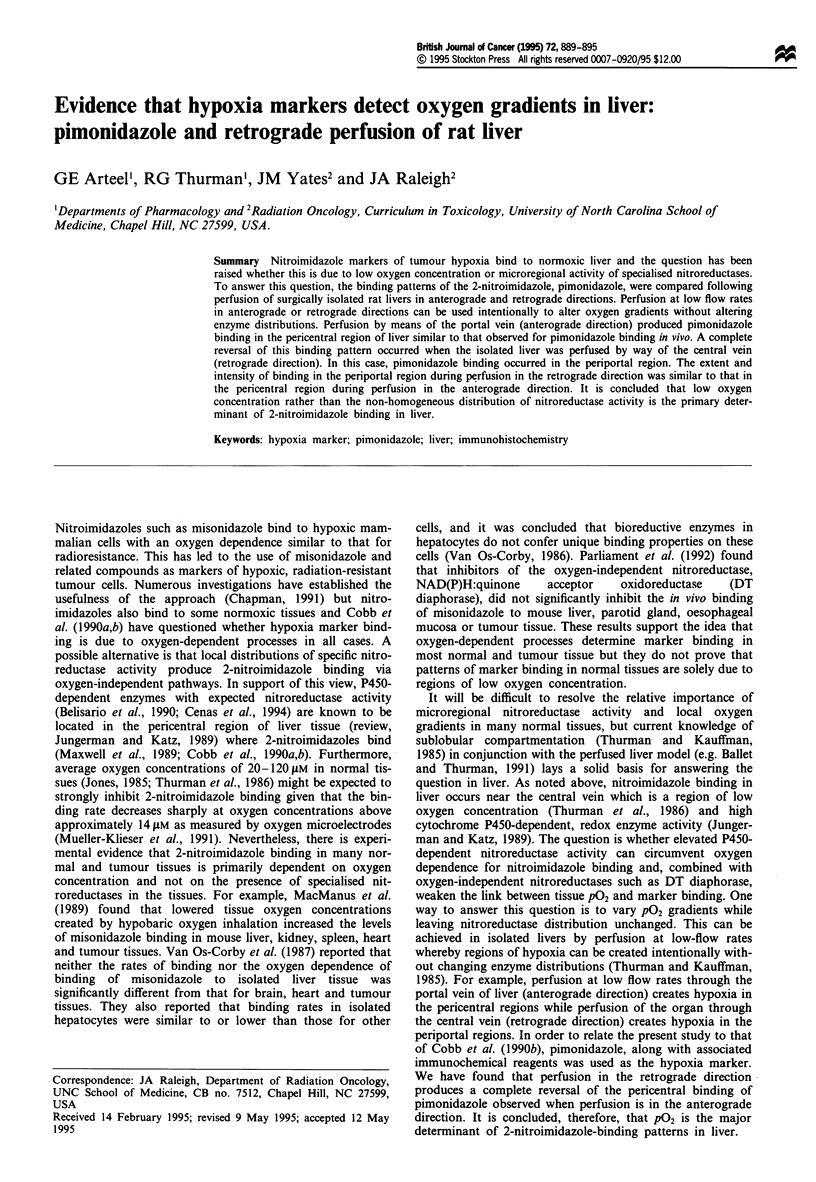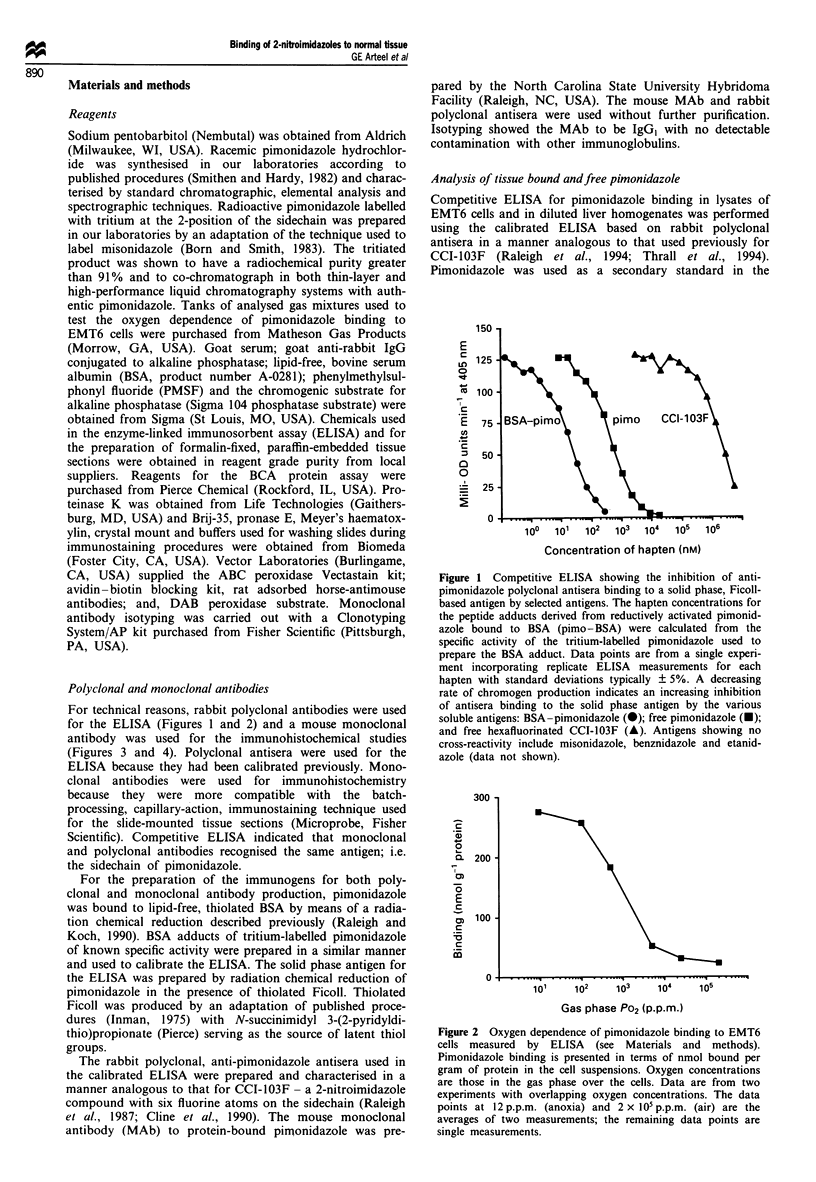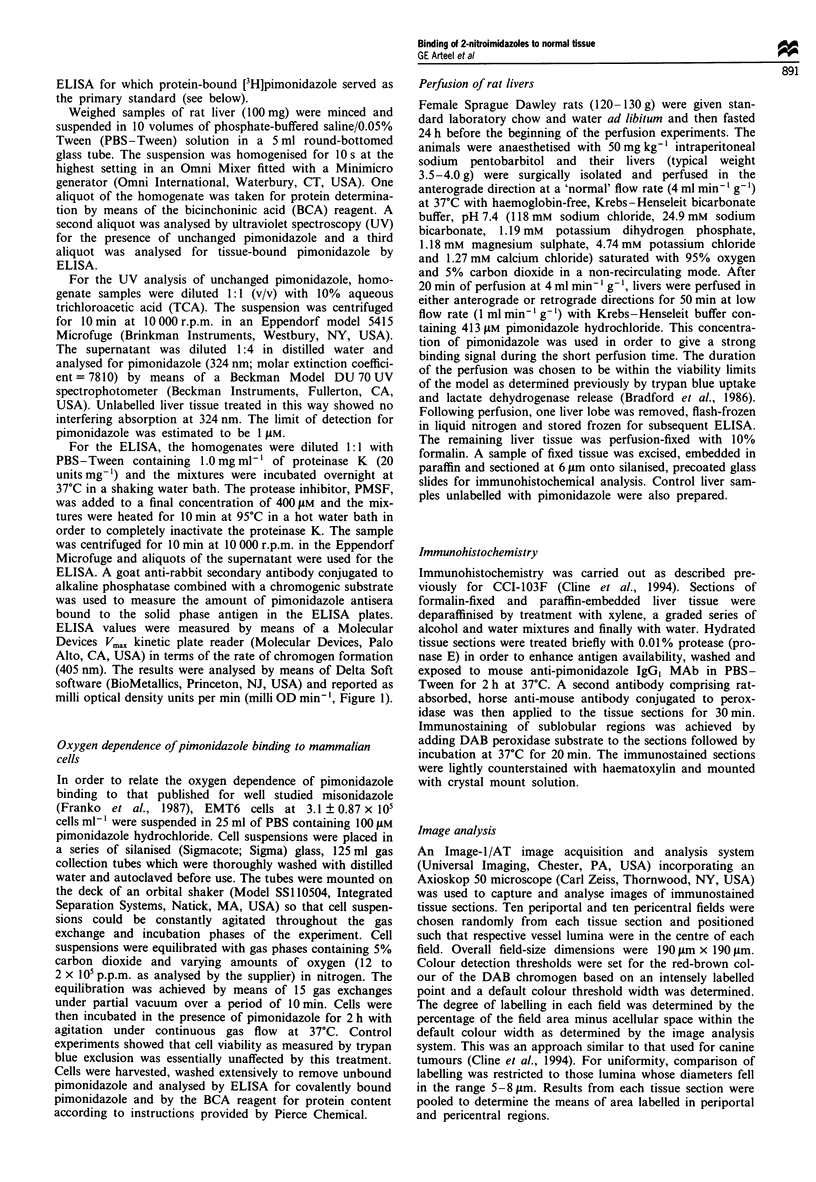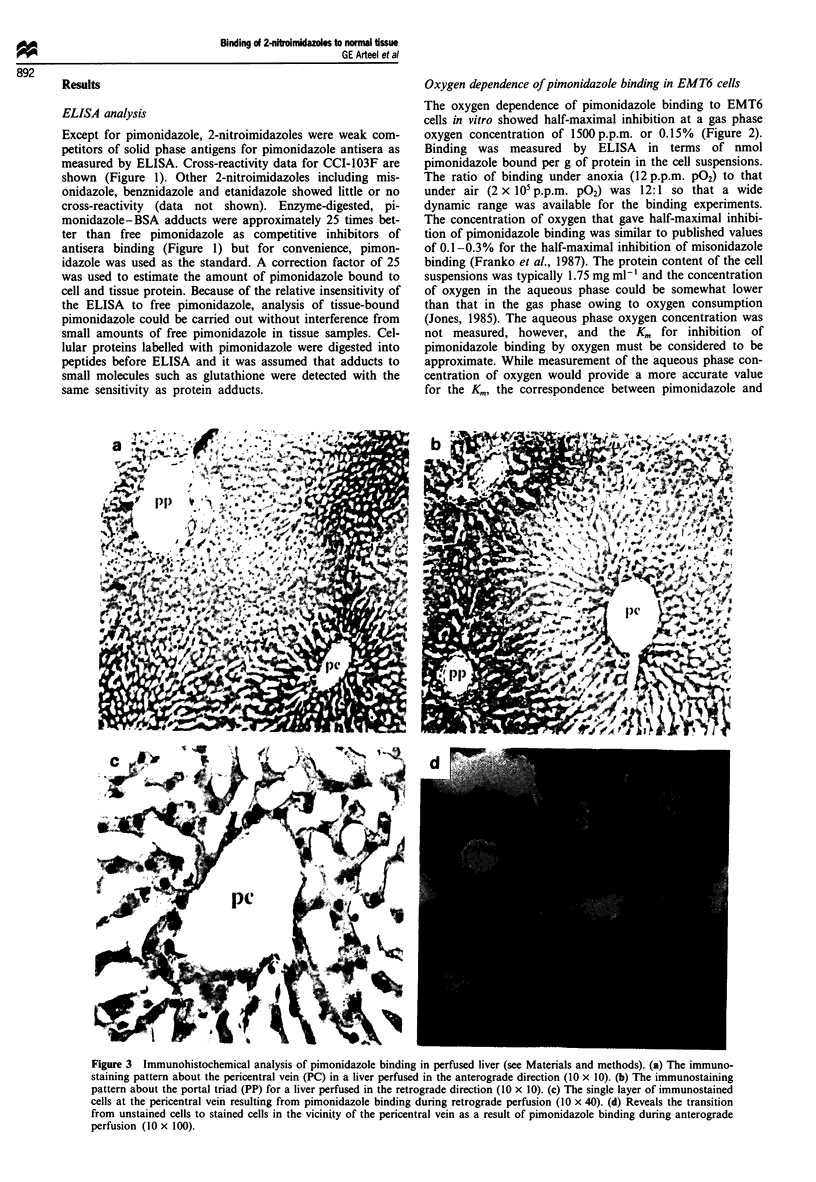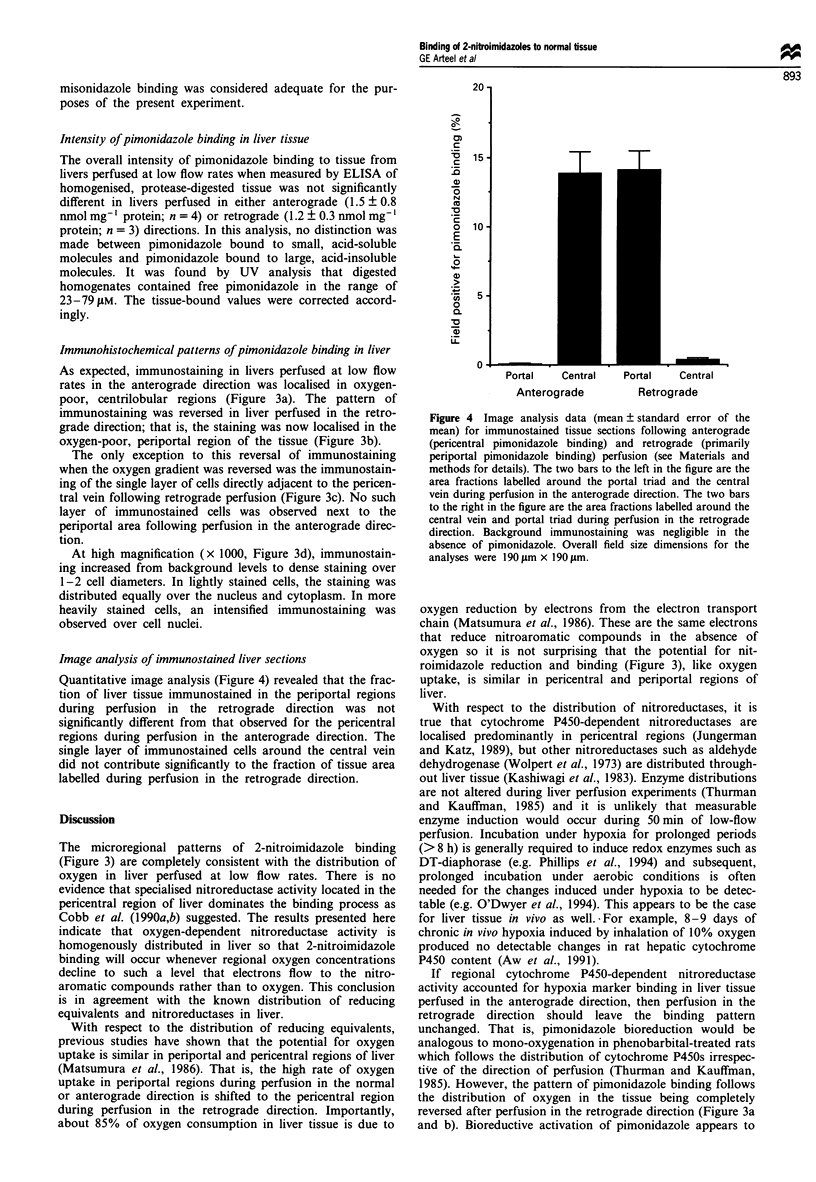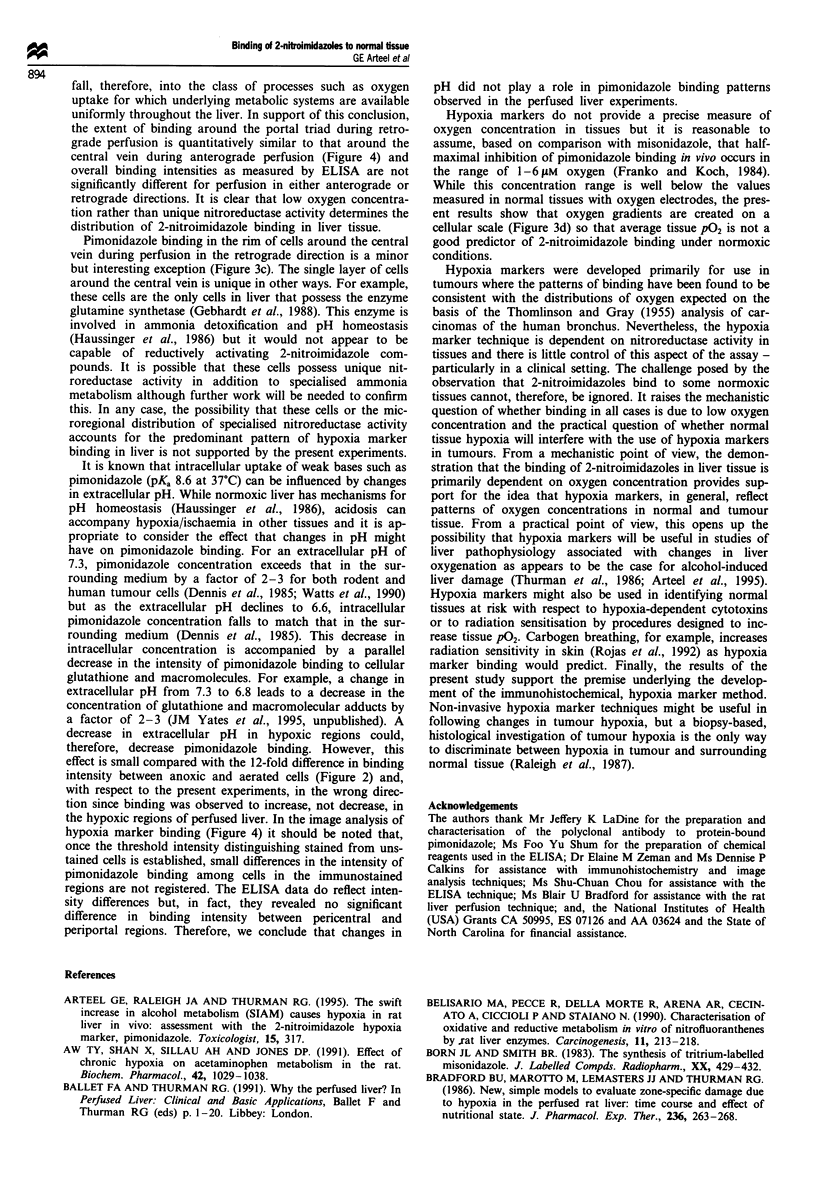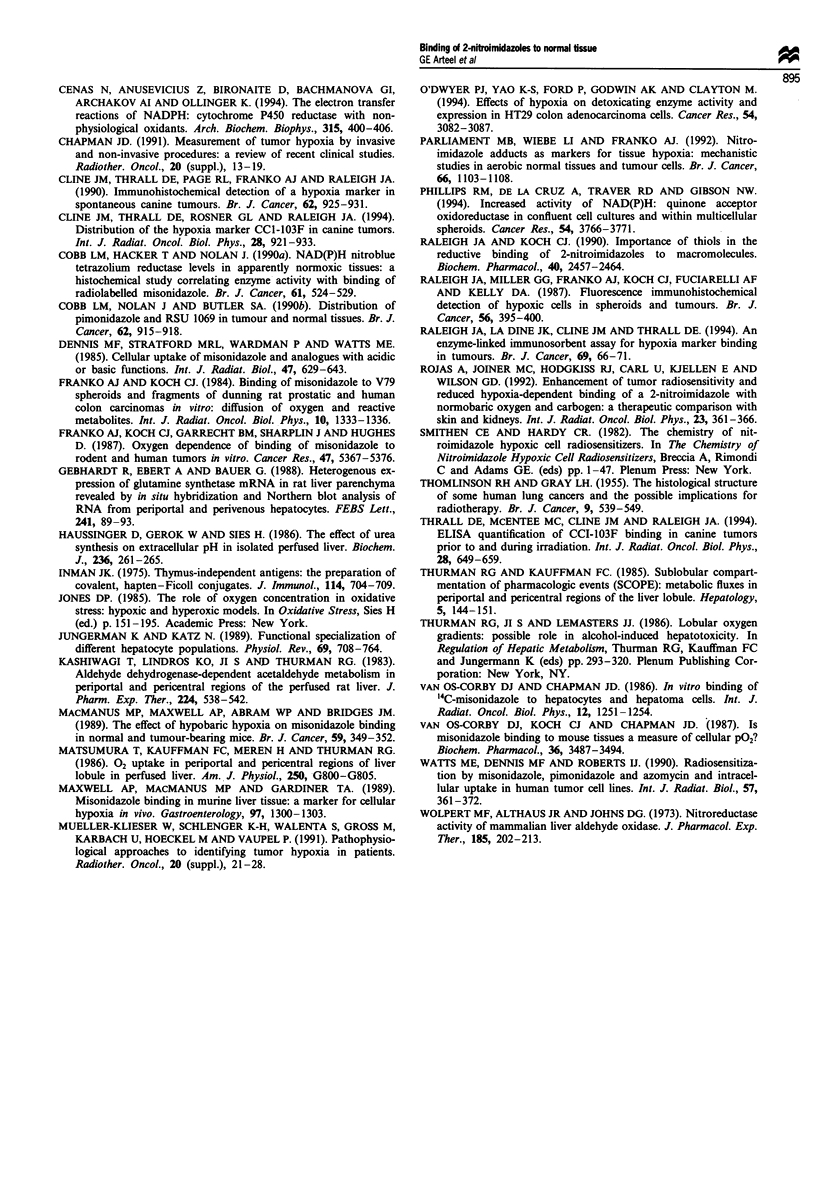# Evidence that hypoxia markers detect oxygen gradients in liver: pimonidazole and retrograde perfusion of rat liver.

**DOI:** 10.1038/bjc.1995.429

**Published:** 1995-10

**Authors:** G. E. Arteel, R. G. Thurman, J. M. Yates, J. A. Raleigh

**Affiliations:** Department of Pharmacology, University of North Carolina School of Medicine, Chapel Hill 27599, USA.

## Abstract

**Images:**


British Journal of Cancer (1995) 72, 889–895
© 1995 Stockton Press All rights reserved 0007–0920/95 $12.00

### Evidence that hypoxia markers detect oxygen gradients in liver: pimonidazole and retrograde perfusion of rat liver

GE Arteel[1], RG Thurman[1], JM Yates[2] and JA Raleigh[2]

[1]*Departments of Pharmacology and* [2]*Radiation Oncology, Curriculum in Toxicology, University of North Carolina School of Medicine, Chapel Hill, NC 27599, USA.*

**Summary** Nitroimidazole markers of tumour hypoxia bind to normoxic liver and the question has been raised whether this is due to low oxygen concentration or microregional activity of specialised nitroreductases. To answer this question, the binding patterns of the 2-nitroimidazole, pimonidazole, were compared following perfusion of surgically isolated rat livers in anterograde and retrograde directions. Perfusion at low flow rates in anterograde or retrograde directions can be used intentionally to alter oxygen gradients without altering enzyme distributions. Perfusion by means of the portal vein (anterograde direction) produced pimonidazole binding in the pericentral region of liver similar to that observed for pimonidazole binding *in vivo*. A complete reversal of this binding pattern occurred when the isolated liver was perfused by way of the central vein (retrograde direction). In this case, pimonidazole binding occurred in the periportal region. The extent and intensity of binding in the periportal region during perfusion in the retrograde direction was similar to that in the pericentral region during perfusion in the anterograde direction. It is concluded that low oxygen concentration rather than the non-homogeneous distribution of nitroreductase activity is the primary determinant of 2-nitroimidazole binding in liver.

**Keywords**: hypoxia marker; pimonidazole; liver; immunohistochemistry

Nitroimidazoles such as misonidazole bind to hypoxic mammalian cells with an oxygen dependence similar to that for radioresistance. This has led to the use of misonidazole and related compounds as markers of hypoxic, radiation-resistant tumour cells. Numerous investigations have established the usefulness of the approach (Chapman, 1991) but nitroimidazoles also bind to some normoxic tissues and Cobb *et al.* (1990*a*,*b*) have questioned whether hypoxia marker binding is due to oxygen-dependent processes in all cases. A possible alternative is that local distributions of specific nitroreductase activity produce 2-nitroimidazole binding via oxygen-independent pathways. In support of this view, P450-dependent enzymes with expected nitroreductase activity (Belisario *et al.*, 1990; Cenas *et al.*, 1994) are known to be located in the pericentral region of liver tissue (review, Jungerman and Katz, 1989) where 2-nitroimidazoles bind (Maxwell *et al.*, 1989; Cobb *et al.*, 1990*a*,*b*). Furthermore, average oxygen concentrations of 20–120 μM in normal tissues (Jones, 1985; Thurman *et al.*, 1986) might be expected to strongly inhibit 2-nitroimidazole binding given that the binding rate decreases sharply at oxygen concentrations above approximately 14 μM as measured by oxygen microelectrodes (Mueller-Klieser *et al.*, 1991). Nevertheless, there is experimental evidence that 2-nitroimidazole binding in many normal and tumour tissues is primarily dependent on oxygen concentration and not on the presence of specialised nitroreductases in the tissues. For example, MacManus *et al.* (1989) found that lowered tissue oxygen concentrations created by hypobaric oxygen inhalation increased the levels of misonidazole binding in mouse liver, kidney, spleen, heart and tumour tissues. Van Os-Corby *et al.* (1987) reported that neither the rates of binding nor the oxygen dependence of binding of misonidazole to isolated liver tissue was significantly different from that for brain, heart and tumour tissues. They also reported that binding rates in isolated hepatocytes were similar to or lower than those for other cells, and it was concluded that bioreductive enzymes in hepatocytes do not confer unique binding properties on these cells (Van Os-Corby, 1986). Parliament *et al.* (1992) found that inhibitors of the oxygen-independent nitroreductase, NAD(P)H:quinone acceptor oxidoreductase (DT diaphorase), did not significantly inhibit the *in vivo* binding of misonidazole to mouse liver, parotid gland, oesophageal mucosa or tumour tissue. These results support the idea that oxygen-dependent processes determine marker binding in most normal and tumour tissue but they do not prove that patterns of marker binding in normal tissues are solely due to regions of low oxygen concentration.

It will be difficult to resolve the relative importance of microregional nitroreductase activity and local oxygen gradients in many normal tissues, but current knowledge of sublobular compartmentation (Thurman and Kauffman, 1985) in conjunction with the perfused liver model (e.g. Ballet and Thurman, 1991) lays a solid basis for answering the question in liver. As noted above, nitroimidazole binding in liver occurs near the central vein which is a region of low oxygen concentration (Thurman *et al.*, 1986) and high cytochrome P450-dependent, redox enzyme activity (Jungerman and Katz, 1989). The question is whether elevated P450-dependent nitroreductase activity can circumvent oxygen dependence for nitroimidazole binding and, combined with oxygen-independent nitroreductases such as DT diaphorase, weaken the link between tissue $pO_2$ and marker binding. One way to answer this question is to vary $pO_2$ gradients while leaving nitroreductase distribution unchanged. This can be achieved in isolated livers by perfusion at low-flow rates whereby regions of hypoxia can be created intentionally without changing enzyme distributions (Thurman and Kauffman, 1985). For example, perfusion at low flow rates through the portal vein of liver (anterograde direction) creates hypoxia in the pericentral regions while perfusion of the organ through the central vein (retrograde direction) creates hypoxia in the periportal regions. In order to relate the present study to that of Cobb *et al.* (1990*b*), pimonidazole, along with associated immunochemical reagents was used as the hypoxia marker. We have found that perfusion in the retrograde direction produces a complete reversal of the pericentral binding of pimonidazole observed when perfusion is in the anterograde direction. It is concluded, therefore, that $pO_2$ is the major determinant of 2-nitroimidazole-binding patterns in liver.

Correspondence: JA Raleigh, Department of Radiation Oncology, UNC School of Medicine, CB no. 7512, Chapel Hill, NC 27599, USA
Received 14 February 1995; revised 9 May 1995; accepted 12 May 1995

### Materials and methods

### Reagents

Sodium pentobarbitol (Nembutal) was obtained from Aldrich (Milwaukee, WI, USA). Racemic pimonidazole hydrochloride was synthesised in our laboratories according to published procedures (Smithen and Hardy, 1982) and characterised by standard chromatographic, elemental analysis and spectrographic techniques. Radioactive pimonidazole labelled with tritium at the 2-position of the sidechain was prepared in our laboratories by an adaptation of the technique used to label misonidazole (Born and Smith, 1983). The tritiated product was shown to have a radiochemical purity greater than 91% and to co-chromatograph in both thin-layer and high-performance liquid chromatography systems with authentic pimonidazole. Tanks of analysed gas mixtures used to test the oxygen dependence of pimonidazole binding to EMT6 cells were purchased from Matheson Gas Products (Morrow, GA, USA). Goat serum; goat anti-rabbit IgG conjugated to alkaline phosphatase; lipid-free, bovine serum albumin (BSA, product number A-0281); phenylmethylsulphonyl fluoride (PMSF) and the chromogenic substrate for alkaline phosphatase (Sigma 104 phosphatase substrate) were obtained from Sigma (St Louis, MO, USA). Chemicals used in the enzyme-linked immunosorbent assay (ELISA) and for the preparation of formalin-fixed, paraffin-embedded tissue sections were obtained in reagent grade purity from local suppliers. Reagents for the BCA protein assay were purchased from Pierce Chemical (Rockford, IL, USA). Proteinase K was obtained from Life Technologies (Gaithersburg, MD, USA) and Brij-35, pronase E, Meyer's haematoxylin, crystal mount and buffers used for washing slides during immunostaining procedures were obtained from Biomeda (Foster City, CA, USA). Vector Laboratories (Burlingame, CA, USA) supplied the ABC peroxidase Vectastain kit; avidin-biotin blocking kit, rat adsorbed horse-antimouse antibodies; and, DAB peroxidase substrate. Monoclonal antibody isotyping was carried out with a Clonotyping System/AP kit purchased from Fisher Scientific (Pittsburgh, PA, USA).

### Polyclonal and monoclonal antibodies

For technical reasons, rabbit polyclonal antibodies were used for the ELISA (Figures 1 and 2) and a mouse monoclonal antibody was used for the immunohistochemical studies (Figures 3 and 4). Polyclonal antisera were used for the ELISA because they had been calibrated previously. Monoclonal antibodies were used for immunohistochemistry because they were more compatible with the batch-processing, capillary-action, immunostaining technique used for the slide-mounted tissue sections (Microprobe, Fisher Scientific). Competitive ELISA indicated that monoclonal and polyclonal antibodies recognised the same antigen; i.e. the sidechain of pimonidazole.

For the preparation of the immunogens for both polyclonal and monoclonal antibody production, pimonidazole was bound to lipid-free, thiolated BSA by means of a radiation chemical reduction described previously (Raleigh and Koch, 1990). BSA adducts of tritium-labelled pimonidazole of known specific activity were prepared in a similar manner and used to calibrate the ELISA. The solid phase antigen for the ELISA was prepared by radiation chemical reduction of pimonidazole in the presence of thiolated Ficoll. Thiolated Ficoll was produced by an adaptation of published procedures (Inman, 1975) with *N*-succinimidyl 3-(2-pyridyldithio)propionate (Pierce) serving as the source of latent thiol groups.

The rabbit polyclonal, anti-pimonidazole antisera used in the calibrated ELISA were prepared and characterised in a manner analogous to that for CCI-103F – a 2-nitroimidazole compound with six fluorine atoms on the sidechain (Raleigh *et al.*, 1987; Cline *et al.*, 1990). The mouse monoclonal antibody (MAb) to protein-bound pimonidazole was prepared by the North Carolina State University Hybridoma Facility (Raleigh, NC, USA). The mouse MAb and rabbit polyclonal antisera were used without further purification. Isotyping showed the MAb to be $IgG_1$ with no detectable contamination with other immunoglobulins.

### Analysis of tissue bound and free pimonidazole

Competitive ELISA for pimonidazole binding in lysates of EMT6 cells and in diluted liver homogenates was performed using the calibrated ELISA based on rabbit polyclonal antisera in a manner analogous to that used previously for CCI-103F (Raleigh *et al.*, 1994; Thrall *et al.*, 1994). Pimonidazole was used as a secondary standard in the

**Figure 1** Competitive ELISA showing the inhibition of anti-pimonidazole polyclonal antisera binding to a solid phase, Ficoll-based antigen by selected antigens. The hapten concentrations for the peptide adducts derived from reductively activated pimonidazole bound to BSA (pimo-BSA) were calculated from the specific activity of the tritium-labelled pimonidazole used to prepare the BSA adduct. Data points are from a single experiment incorporating replicate ELISA measurements for each hapten with standard deviations typically ± 5%. A decreasing rate of chromogen production indicates an increasing inhibition of antisera binding to the solid phase antigen by the various soluble antigens: BSA-pimonidazole (●); free pimonidazole (■); and free hexafluorinated CCI-103F (▲). Antigens showing no cross-reactivity include misonidazole, benznidazole and etanidazole (data not shown).

**Figure 2** Oxygen dependence of pimonidazole binding to EMT6 cells measured by ELISA (see Materials and methods). Pimonidazole binding is presented in terms of nmol bound per gram of protein in the cell suspensions. Oxygen concentrations are those in the gas phase over the cells. Data are from two experiments with overlapping oxygen concentrations. The data points at 12 p.p.m. (anoxia) and $2 \times 10^5$ p.p.m. (air) are the averages of two measurements; the remaining data points are single measurements.

ELISA for which protein-bound [$^3$H]pimonidazole served as the primary standard (see below).

Weighed samples of rat liver (100 mg) were minced and suspended in 10 volumes of phosphate-buffered saline/0.05% Tween (PBS–Tween) solution in a 5 ml round-bottomed glass tube. The suspension was homogenised for 10 s at the highest setting in an Omni Mixer fitted with a Minimicro generator (Omni International, Waterbury, CT, USA). One aliquot of the homogenate was taken for protein determination by means of the bicinchoninic acid (BCA) reagent. A second aliquot was analysed by ultraviolet spectroscopy (UV) for the presence of unchanged pimonidazole and a third aliquot was analysed for tissue-bound pimonidazole by ELISA.

For the UV analysis of unchanged pimonidazole, homogenate samples were diluted 1:1 (v/v) with 10% aqueous trichloroacetic acid (TCA). The suspension was centrifuged for 10 min at 10 000 r.p.m. in an Eppendorf model 5415 Microfuge (Brinkman Instruments, Westbury, NY, USA). The supernatant was diluted 1:4 in distilled water and analysed for pimonidazole (324 nm; molar extinction coefficient = 7810) by means of a Beckman Model DU 70 UV spectrophotometer (Beckman Instruments, Fullerton, CA, USA). Unlabelled liver tissue treated in this way showed no interfering absorption at 324 nm. The limit of detection for pimonidazole was estimated to be 1 $\mu$M.

For the ELISA, the homogenates were diluted 1:1 with PBS–Tween containing 1.0 mg ml$^{-1}$ of proteinase K (20 units mg$^{-1}$) and the mixtures were incubated overnight at 37°C in a shaking water bath. The protease inhibitor, PMSF, was added to a final concentration of 400 $\mu$M and the mixtures were heated for 10 min at 95°C in a hot water bath in order to completely inactivate the proteinase K. The sample was centrifuged for 10 min at 10 000 r.p.m. in the Eppendorf Microfuge and aliquots of the supernatant were used for the ELISA. A goat anti-rabbit secondary antibody conjugated to alkaline phosphatase combined with a chromogenic substrate was used to measure the amount of pimonidazole antisera bound to the solid phase antigen in the ELISA plates. ELISA values were measured by means of a Molecular Devices $V_{max}$ kinetic plate reader (Molecular Devices, Palo Alto, CA, USA) in terms of the rate of chromogen formation (405 nm). The results were analysed by means of Delta Soft software (BioMetallics, Princeton, NJ, USA) and reported as milli optical density units per min (milli OD min$^{-1}$, Figure 1).

### *Oxygen dependence of pimonidazole binding to mammalian cells*

In order to relate the oxygen dependence of pimonidazole binding to that published for well studied misonidazole (Franko *et al.*, 1987), EMT6 cells at $3.1 \pm 0.87 \times 10^5$ cells ml$^{-1}$ were suspended in 25 ml of PBS containing 100 $\mu$M pimonidazole hydrochloride. Cell suspensions were placed in a series of silanised (Sigmacote; Sigma) glass, 125 ml gas collection tubes which were thoroughly washed with distilled water and autoclaved before use. The tubes were mounted on the deck of an orbital shaker (Model SS110504, Integrated Separation Systems, Natick, MA, USA) so that cell suspensions could be constantly agitated throughout the gas exchange and incubation phases of the experiment. Cell suspensions were equilibrated with gas phases containing 5% carbon dioxide and varying amounts of oxygen (12 to $2 \times 10^5$ p.p.m. as analysed by the supplier) in nitrogen. The equilibration was achieved by means of 15 gas exchanges under partial vacuum over a period of 10 min. Cells were then incubated in the presence of pimonidazole for 2 h with agitation under continuous gas flow at 37°C. Control experiments showed that cell viability as measured by trypan blue exclusion was essentially unaffected by this treatment. Cells were harvested, washed extensively to remove unbound pimonidazole and analysed by ELISA for covalently bound pimonidazole and by the BCA reagent for protein content according to instructions provided by Pierce Chemical.

### *Perfusion of rat livers*

Female Sprague Dawley rats (120–130 g) were given standard laboratory chow and water *ad libitum* and then fasted 24 h before the beginning of the perfusion experiments. The animals were anaesthetised with 50 mg kg$^{-1}$ intraperitoneal sodium pentobarbitol and their livers (typical weight 3.5–4.0 g) were surgically isolated and perfused in the anterograde direction at a 'normal' flow rate (4 ml min$^{-1}$ g$^{-1}$) at 37°C with haemoglobin-free, Krebs–Henseleit bicarbonate buffer, pH 7.4 (118 mM sodium chloride, 24.9 mM sodium bicarbonate, 1.19 mM potassium dihydrogen phosphate, 1.18 mM magnesium sulphate, 4.74 mM potassium chloride and 1.27 mM calcium chloride) saturated with 95% oxygen and 5% carbon dioxide in a non-recirculating mode. After 20 min of perfusion at 4 ml min$^{-1}$ g$^{-1}$, livers were perfused in either anterograde or retrograde directions for 50 min at low flow rate (1 ml min$^{-1}$ g$^{-1}$) with Krebs–Henseleit buffer containing 413 $\mu$M pimonidazole hydrochloride. This concentration of pimonidazole was used in order to give a strong binding signal during the short perfusion time. The duration of the perfusion was chosen to be within the viability limits of the model as determined previously by trypan blue uptake and lactate dehydrogenase release (Bradford *et al.*, 1986). Following perfusion, one liver lobe was removed, flash-frozen in liquid nitrogen and stored frozen for subsequent ELISA. The remaining liver tissue was perfusion-fixed with 10% formalin. A sample of fixed tissue was excised, embedded in paraffin and sectioned at 6 $\mu$m onto silanised, precoated glass slides for immunohistochemical analysis. Control liver samples unlabelled with pimonidazole were also prepared.

### *Immunohistochemistry*

Immunohistochemistry was carried out as described previously for CCI-103F (Cline *et al.*, 1994). Sections of formalin-fixed and paraffin-embedded liver tissue were deparaffinised by treatment with xylene, a graded series of alcohol and water mixtures and finally with water. Hydrated tissue sections were treated briefly with 0.01% protease (pronase E) in order to enhance antigen availability, washed and exposed to mouse anti-pimonidazole $IgG_1$ MAb in PBS–Tween for 2 h at 37°C. A second antibody comprising rat-absorbed, horse anti-mouse antibody conjugated to peroxidase was then applied to the tissue sections for 30 min. Immunostaining of sublobular regions was achieved by adding DAB peroxidase substrate to the sections followed by incubation at 37°C for 20 min. The immunostained sections were lightly counterstained with haematoxylin and mounted with crystal mount solution.

### *Image analysis*

An Image-1/AT image acquisition and analysis system (Universal Imaging, Chester, PA, USA) incorporating an Axioskop 50 microscope (Carl Zeiss, Thornwood, NY, USA) was used to capture and analyse images of immunostained tissue sections. Ten periportal and ten pericentral fields were chosen randomly from each tissue section and positioned such that respective vessel lumina were in the centre of each field. Overall field-size dimensions were 190 $\mu$m × 190 $\mu$m. Colour detection thresholds were set for the red-brown colour of the DAB chromogen based on an intensely labelled point and a default colour threshold width was determined. The degree of labelling in each field was determined by the percentage of the field area minus acellular space within the default colour width as determined by the image analysis system. This was an approach similar to that used for canine tumours (Cline *et al.*, 1994). For uniformity, comparison of labelling was restricted to those lumina whose diameters fell in the range 5–8 $\mu$m. Results from each tissue section were pooled to determine the means of area labelled in periportal and pericentral regions.

### Results

### ELISA analysis

Except for pimonidazole, 2-nitroimidazoles were weak competitors of solid phase antigens for pimonidazole antisera as measured by ELISA. Cross-reactivity data for CCI-103F are shown (Figure 1). Other 2-nitroimidazoles including misonidazole, benznidazole and etanidazole showed little or no cross-reactivity (data not shown). Enzyme-digested, pimonidazole-BSA adducts were approximately 25 times better than free pimonidazole as competitive inhibitors of antisera binding (Figure 1) but for convenience, pimonidazole was used as the standard. A correction factor of 25 was used to estimate the amount of pimonidazole bound to cell and tissue protein. Because of the relative insensitivity of the ELISA to free pimonidazole, analysis of tissue-bound pimonidazole could be carried out without interference from small amounts of free pimonidazole in tissue samples. Cellular proteins labelled with pimonidazole were digested into peptides before ELISA and it was assumed that adducts to small molecules such as glutathione were detected with the same sensitivity as protein adducts.

### Oxygen dependence of pimonidazole binding in EMT6 cells

The oxygen dependence of pimonidazole binding to EMT6 cells *in vitro* showed half-maximal inhibition at a gas phase oxygen concentration of 1500 p.p.m. or 0.15% (Figure 2). Binding was measured by ELISA in terms of nmol pimonidazole bound per g of protein in the cell suspensions. The ratio of binding under anoxia (12 p.p.m. $pO_2$) to that under air ($2 \times 10^5$ p.p.m. $pO_2$) was 12:1 so that a wide dynamic range was available for the binding experiments. The concentration of oxygen that gave half-maximal inhibition of pimonidazole binding was similar to published values of 0.1–0.3% for the half-maximal inhibition of misonidazole binding (Franko *et al.*, 1987). The protein content of the cell suspensions was typically 1.75 mg $ml^{-1}$ and the concentration of oxygen in the aqueous phase could be somewhat lower than that in the gas phase owing to oxygen consumption (Jones, 1985). The aqueous phase oxygen concentration was not measured, however, and the $K_m$ for inhibition of pimonidazole binding by oxygen must be considered to be approximate. While measurement of the aqueous phase concentration of oxygen would provide a more accurate value for the $K_m$, the correspondence between pimonidazole and

**Figure 3** Immunohistochemical analysis of pimonidazole binding in perfused liver (see Materials and methods). (**a**) The immunostaining pattern about the pericentral vein (PC) in a liver perfused in the anterograde direction (10 × 10). (**b**) The immunostaining pattern about the portal triad (PP) for a liver perfused in the retrograde direction (10 × 10). (**c**) The single layer of immunostained cells at the pericentral vein resulting from pimonidazole binding during retrograde perfusion (10 × 40). (**d**) Reveals the transition from unstained cells to stained cells in the vicinity of the pericentral vein as a result of pimonidazole binding during anterograde perfusion (10 × 100).

misonidazole binding was considered adequate for the purposes of the present experiment.

*Intensity of pimonidazole binding in liver tissue*

The overall intensity of pimonidazole binding to tissue from livers perfused at low flow rates when measured by ELISA of homogenised, protease-digested tissue was not significantly different in livers perfused in either anterograde ($1.5 \pm 0.8$ nmol $mg^{-1}$ protein; $n = 4$) or retrograde ($1.2 \pm 0.3$ nmol $mg^{-1}$ protein; $n = 3$) directions. In this analysis, no distinction was made between pimonidazole bound to small, acid-soluble molecules and pimonidazole bound to large, acid-insoluble molecules. It was found by UV analysis that digested homogenates contained free pimonidazole in the range of 23–79 μM. The tissue-bound values were corrected accordingly.

*Immunohistochemical patterns of pimonidazole binding in liver*

As expected, immunostaining in livers perfused at low flow rates in the anterograde direction was localised in oxygen-poor, centrilobular regions (Figure 3a). The pattern of immunostaining was reversed in liver perfused in the retrograde direction; that is, the staining was now localised in the oxygen-poor, periportal region of the tissue (Figure 3b).

The only exception to this reversal of immunostaining when the oxygen gradient was reversed was the immunostaining of the single layer of cells directly adjacent to the pericentral vein following retrograde perfusion (Figure 3c). No such layer of immunostained cells was observed next to the periportal area following perfusion in the anterograde direction.

At high magnification (× 1000, Figure 3d), immunostaining increased from background levels to dense staining over 1–2 cell diameters. In lightly stained cells, the staining was distributed equally over the nucleus and cytoplasm. In more heavily stained cells, an intensified immunostaining was observed over cell nuclei.

*Image analysis of immunostained liver sections*

Quantitative image analysis (Figure 4) revealed that the fraction of liver tissue immunostained in the periportal regions during perfusion in the retrograde direction was not significantly different from that observed for the pericentral regions during perfusion in the anterograde direction. The single layer of immunostained cells around the central vein did not contribute significantly to the fraction of tissue area labelled during perfusion in the retrograde direction.

**Discussion**

The microregional patterns of 2-nitroimidazole binding (Figure 3) are completely consistent with the distribution of oxygen in liver perfused at low flow rates. There is no evidence that specialised nitroreductase activity located in the pericentral region of liver dominates the binding process as Cobb *et al.* (1990*a*,*b*) suggested. The results presented here indicate that oxygen-dependent nitroreductase activity is homogenously distributed in liver so that 2-nitroimidazole binding will occur whenever regional oxygen concentrations decline to such a level that electrons flow to the nitroaromatic compounds rather than to oxygen. This conclusion is in agreement with the known distribution of reducing equivalents and nitroreductases in liver.

With respect to the distribution of reducing equivalents, previous studies have shown that the potential for oxygen uptake is similar in periportal and pericentral regions of liver (Matsumura *et al.*, 1986). That is, the high rate of oxygen uptake in periportal regions during perfusion in the normal or anterograde direction is shifted to the pericentral region during perfusion in the retrograde direction. Importantly, about 85% of oxygen consumption in liver tissue is due to

**Figure 4** Image analysis data (mean ± standard error of the mean) for immunostained tissue sections following anterograde (pericentral pimonidazole binding) and retrograde (primarily periportal pimonidazole binding) perfusion (see Materials and methods for details). The two bars to the left in the figure are the area fractions labelled around the portal triad and the central vein during perfusion in the anterograde direction. The two bars to the right in the figure are the area fractions labelled around the central vein and portal triad during perfusion in the retrograde direction. Background immunostaining was negligible in the absence of pimonidazole. Overall field size dimensions for the analyses were 190 μm × 190 μm.

oxygen reduction by electrons from the electron transport chain (Matsumura *et al.*, 1986). These are the same electrons that reduce nitroaromatic compounds in the absence of oxygen so it is not surprising that the potential for nitroimidazole reduction and binding (Figure 3), like oxygen uptake, is similar in pericentral and periportal regions of liver.

With respect to the distribution of nitroreductases, it is true that cytochrome P450-dependent nitroreductases are localised predominantly in pericentral regions (Jungerman and Katz, 1989), but other nitroreductases such as aldehyde dehydrogenase (Wolpert *et al.*, 1973) are distributed throughout liver tissue (Kashiwagi *et al.*, 1983). Enzyme distributions are not altered during liver perfusion experiments (Thurman and Kauffman, 1985) and it is unlikely that measurable enzyme induction would occur during 50 min of low-flow perfusion. Incubation under hypoxia for prolonged periods (> 8 h) is generally required to induce redox enzymes such as DT-diaphorase (e.g. Phillips *et al.*, 1994) and subsequent, prolonged incubation under aerobic conditions is often needed for the changes induced under hypoxia to be detectable (e.g. O'Dwyer *et al.*, 1994). This appears to be the case for liver tissue *in vivo* as well. For example, 8–9 days of chronic *in vivo* hypoxia induced by inhalation of 10% oxygen produced no detectable changes in rat hepatic cytochrome P450 content (Aw *et al.*, 1991).

If regional cytochrome P450-dependent nitroreductase activity accounted for hypoxia marker binding in liver tissue perfused in the anterograde direction, then perfusion in the retrograde direction should leave the binding pattern unchanged. That is, pimonidazole bioreduction would be analogous to mono-oxygenation in phenobarbital-treated rats which follows the distribution of cytochrome P450s irrespective of the direction of perfusion (Thurman and Kauffman, 1985). However, the pattern of pimonidazole binding follows the distribution of oxygen in the tissue being completely reversed after perfusion in the retrograde direction (Figure 3a and b). Bioreductive activation of pimonidazole appears to fall, therefore, into the class of processes such as oxygen uptake for which underlying metabolic systems are available uniformly throughout the liver. In support of this conclusion, the extent of binding around the portal triad during retrograde perfusion is quantitatively similar to that around the central vein during anterograde perfusion (Figure 4) and overall binding intensities as measured by ELISA are not significantly different for perfusion in either anterograde or retrograde directions. It is clear that low oxygen concentration rather than unique nitroreductase activity determines the distribution of 2-nitroimidazole binding in liver tissue.

Pimonidazole binding in the rim of cells around the central vein during perfusion in the retrograde direction is a minor but interesting exception (Figure 3c). The single layer of cells around the central vein is unique in other ways. For example, these cells are the only cells in liver that possess the enzyme glutamine synthetase (Gebhardt *et al.*, 1988). This enzyme is involved in ammonia detoxification and pH homeostasis (Haussinger *et al.*, 1986) but it would not appear to be capable of reductively activating 2-nitroimidazole compounds. It is possible that these cells possess unique nitroreductase activity in addition to specialised ammonia metabolism although further work will be needed to confirm this. In any case, the possibility that these cells or the microregional distribution of specialised nitroreductase activity accounts for the predominant pattern of hypoxia marker binding in liver is not supported by the present experiments.

It is known that intracellular uptake of weak bases such as pimonidazole ($pK_a$ 8.6 at 37°C) can be influenced by changes in extracellular pH. While normoxic liver has mechanisms for pH homeostasis (Haussinger *et al.*, 1986), acidosis can accompany hypoxia/ischaemia in other tissues and it is appropriate to consider the effect that changes in pH might have on pimonidazole binding. For an extracellular pH of 7.3, pimonidazole concentration exceeds that in the surrounding medium by a factor of 2–3 for both rodent and human tumour cells (Dennis *et al.*, 1985; Watts *et al.*, 1990) but as the extracellular pH declines to 6.6, intracellular pimonidazole concentration falls to match that in the surrounding medium (Dennis *et al.*, 1985). This decrease in intracellular concentration is accompanied by a parallel decrease in the intensity of pimonidazole binding to cellular glutathione and macromolecules. For example, a change in extracellular pH from 7.3 to 6.8 leads to a decrease in the concentration of glutathione and macromolecular adducts by a factor of 2–3 (JM Yates *et al.*, 1995, unpublished). A decrease in extracellular pH in hypoxic regions could, therefore, decrease pimonidazole binding. However, this effect is small compared with the 12-fold difference in binding intensity between anoxic and aerated cells (Figure 2) and, with respect to the present experiments, in the wrong direction since binding was observed to increase, not decrease, in the hypoxic regions of perfused liver. In the image analysis of hypoxia marker binding (Figure 4) it should be noted that, once the threshold intensity distinguishing stained from unstained cells is established, small differences in the intensity of pimonidazole binding among cells in the immunostained regions are not registered. The ELISA data do reflect intensity differences but, in fact, they revealed no significant difference in binding intensity between pericentral and periportal regions. Therefore, we conclude that changes in pH did not play a role in pimonidazole binding patterns observed in the perfused liver experiments.

Hypoxia markers do not provide a precise measure of oxygen concentration in tissues but it is reasonable to assume, based on comparison with misonidazole, that half-maximal inhibition of pimonidazole binding *in vivo* occurs in the range of 1–6 μM oxygen (Franko and Koch, 1984). While this concentration range is well below the values measured in normal tissues with oxygen electrodes, the present results show that oxygen gradients are created on a cellular scale (Figure 3d) so that average tissue $pO_2$ is not a good predictor of 2-nitroimidazole binding under normoxic conditions.

Hypoxia markers were developed primarily for use in tumours where the patterns of binding have been found to be consistent with the distributions of oxygen expected on the basis of the Thomlinson and Gray (1955) analysis of carcinomas of the human bronchus. Nevertheless, the hypoxia marker technique is dependent on nitroreductase activity in tissues and there is little control of this aspect of the assay – particularly in a clinical setting. The challenge posed by the observation that 2-nitroimidazoles bind to some normoxic tissues cannot, therefore, be ignored. It raises the mechanistic question of whether binding in all cases is due to low oxygen concentration and the practical question of whether normal tissue hypoxia will interfere with the use of hypoxia markers in tumours. From a mechanistic point of view, the demonstration that the binding of 2-nitroimidazoles in liver tissue is primarily dependent on oxygen concentration provides support for the idea that hypoxia markers, in general, reflect patterns of oxygen concentrations in normal and tumour tissue. From a practical point of view, this opens up the possibility that hypoxia markers will be useful in studies of liver pathophysiology associated with changes in liver oxygenation as appears to be the case for alcohol-induced liver damage (Thurman *et al.*, 1986; Arteel *et al.*, 1995). Hypoxia markers might also be used in identifying normal tissues at risk with respect to hypoxia-dependent cytotoxins or to radiation sensitisation by procedures designed to increase tissue $pO_2$. Carbogen breathing, for example, increases radiation sensitivity in skin (Rojas *et al.*, 1992) as hypoxia marker binding would predict. Finally, the results of the present study support the premise underlying the development of the immunohistochemical, hypoxia marker method. Non-invasive hypoxia marker techniques might be useful in following changes in tumour hypoxia, but a biopsy-based, histological investigation of tumour hypoxia is the only way to discriminate between hypoxia in tumour and surrounding normal tissue (Raleigh *et al.*, 1987).

**Acknowledgements**

The authors thank Mr Jeffery K LaDine for the preparation and characterisation of the polyclonal antibody to protein-bound pimonidazole; Ms Foo Yu Shum for the preparation of chemical reagents used in the ELISA; Dr Elaine M Zeman and Ms Dennise P Calkins for assistance with immunohistochemistry and image analysis techniques; Ms Shu-Chuan Chou for assistance with the ELISA technique; Ms Blair U Bradford for assistance with the rat liver perfusion technique; and, the National Institutes of Health (USA) Grants CA 50995, ES 07126 and AA 03624 and the State of North Carolina for financial assistance.

**References**

ARTEEL GE, RALEIGH JA AND THURMAN RG. (1995). The swift increase in alcohol metabolism (SIAM) causes hypoxia in rat liver in vivo: assessment with the 2-nitroimidazole hypoxia marker, pimonidazole. *Toxicologist*, **15**, 317.

AW TY, SHAN X, SILLAU AH AND JONES DP. (1991). Effect of chronic hypoxia on acetaminophen metabolism in the rat. *Biochem. Pharmacol.*, **42**, 1029–1038.

BALLET FA AND THURMAN RG. (1991). Why the perfused liver? In *Perfused Liver: Clinical and Basic Applications*, Ballet F and Thurman RG (eds) p. 1–20. Libbey: London.

BELISARIO MA, PECCE R, DELLA MORTE R, ARENA AR, CECINATO A, CICCIOLI P AND STAIANO N. (1990). Characterisation of oxidative and reductive metabolism *in vitro* of nitrofluoranthenes by rat liver enzymes. *Carcinogenesis*, **11**, 213–218.

BORN JL AND SMITH BR. (1983). The synthesis of tritrium-labelled misonidazole. *J. Labelled Compds. Radiopharm.*, **XX**, 429–432.

BRADFORD BU, MAROTTO M, LEMASTERS JJ AND THURMAN RG. (1986). New, simple models to evaluate zone-specific damage due to hypoxia in the perfused rat liver: time course and effect of nutritional state. *J. Pharmacol. Exp. Ther.*, **236**, 263–268.

CENAS N, ANUSEVICIUS Z, BIRONAITE D, BACHMANOVA GI, ARCHAKOV AI AND OLLINGER K. (1994). The electron transfer reactions of NADPH: cytochrome P450 reductase with non-physiological oxidants. *Arch. Biochem. Biophys.*, **315**, 400–406.

CHAPMAN JD. (1991). Measurement of tumor hypoxia by invasive and non-invasive procedures: a review of recent clinical studies. *Radiother. Oncol.*, **20** (suppl.), 13–19.

CLINE JM, THRALL DE, PAGE RL, FRANKO AJ AND RALEIGH JA. (1990). Immunohistochemical detection of a hypoxia marker in spontaneous canine tumours. *Br. J. Cancer*, **62**, 925–931.

CLINE JM, THRALL DE, ROSNER GL AND RALEIGH JA. (1994). Distribution of the hypoxia marker CC1-103F in canine tumors. *Int. J. Radiat. Oncol. Biol. Phys.*, **28**, 921–933.

COBB LM, HACKER T AND NOLAN J. (1990*a*). NAD(P)H nitroblue tetrazolium reductase levels in apparently normoxic tissues: a histochemical study correlating enzyme activity with binding of radiolabelled misonidazole. *Br. J. Cancer*, **61**, 524–529.

COBB LM, NOLAN J AND BUTLER SA. (1990*b*). Distribution of pimonidazole and RSU 1069 in tumour and normal tissues. *Br. J. Cancer*, **62**, 915–918.

DENNIS MF, STRATFORD MRL, WARDMAN P AND WATTS ME. (1985). Cellular uptake of misonidazole and analogues with acidic or basic functions. *Int. J. Radiat. Biol.*, **47**, 629–643.

FRANKO AJ AND KOCH CJ. (1984). Binding of misonidazole to V79 spheroids and fragments of dunning rat prostatic and human colon carcinomas *in vitro*: diffusion of oxygen and reactive metabolites. *Int. J. Radiat. Oncol. Biol. Phys.*, **10**, 1333–1336.

FRANKO AJ, KOCH CJ, GARRECHT BM, SHARPLIN J AND HUGHES D. (1987). Oxygen dependence of binding of misonidazole to rodent and human tumors *in vitro*. *Cancer Res.*, **47**, 5367–5376.

GEBHARDT R, EBERT A AND BAUER G. (1988). Heterogenous expression of glutamine synthetase mRNA in rat liver parenchyma revealed by *in situ* hybridization and Northern blot analysis of RNA from periportal and perivenous hepatocytes. *FEBS Lett.*, **241**, 89–93.

HAUSSINGER D, GEROK W AND SIES H. (1986). The effect of urea synthesis on extracellular pH in isolated perfused liver. *Biochem. J.*, **236**, 261–265.

INMAN JK. (1975). Thymus-independent antigens: the preparation of covalent, hapten–Ficoll conjugates. *J. Immunol.*, **114**, 704–709.

JONES DP. (1985). The role of oxygen concentration in oxidative stress: hypoxic and hyperoxic models. In *Oxidative Stress*, Sies H (ed.) p. 151–195. Academic Press: New York.

JUNGERMAN K AND KATZ N. (1989). Functional specialization of different hepatocyte populations. *Physiol. Rev.*, **69**, 708–764.

KASHIWAGI T, LINDROS KO, JI S AND THURMAN RG. (1983). Aldehyde dehydrogenase-dependent acetaldehyde metabolism in periportal and pericentral regions of the perfused rat liver. *J. Pharm. Exp. Ther.*, **224**, 538–542.

MACMANUS MP, MAXWELL AP, ABRAM WP AND BRIDGES JM. (1989). The effect of hypobaric hypoxia on misonidazole binding in normal and tumour-bearing mice. *Br. J. Cancer*, **59**, 349–352.

MATSUMURA T, KAUFFMAN FC, MEREN H AND THURMAN RG. (1986). $O_2$ uptake in periportal and pericentral regions of liver lobule in perfused liver. *Am. J. Physiol.*, **250**, G800–G805.

MAXWELL AP, MACMANUS MP AND GARDINER TA. (1989). Misonidazole binding in murine liver tissue: a marker for cellular hypoxia *in vivo*. *Gastroenterology*, **97**, 1300–1303.

MUELLER-KLIESER W, SCHLENGER K-H, WALENTA S, GROSS M, KARBACH U, HOECKEL M AND VAUPEL P. (1991). Pathophysiological approaches to identifying tumor hypoxia in patients. *Radiother. Oncol.*, **20** (suppl.), 21–28.

O'DWYER PJ, YAO K-S, FORD P, GODWIN AK AND CLAYTON M. (1994). Effects of hypoxia on detoxicating enzyme activity and expression in HT29 colon adenocarcinoma cells. *Cancer Res.*, **54**, 3082–3087.

PARLIAMENT MB, WIEBE LI AND FRANKO AJ. (1992). Nitroimidazole adducts as markers for tissue hypoxia: mechanistic studies in aerobic normal tissues and tumour cells. *Br. J. Cancer*, **66**, 1103–1108.

PHILLIPS RM, DE LA CRUZ A, TRAVER RD AND GIBSON NW. (1994). Increased activity of NAD(P)H: quinone acceptor oxidoreductase in confluent cell cultures and within multicellular spheroids. *Cancer Res.*, **54**, 3766–3771.

RALEIGH JA AND KOCH CJ. (1990). Importance of thiols in the reductive binding of 2-nitroimidazoles to macromolecules. *Biochem. Pharmacol.*, **40**, 2457–2464.

RALEIGH JA, MILLER GG, FRANKO AJ, KOCH CJ, FUCIARELLI AF AND KELLY DA. (1987). Fluorescence immunohistochemical detection of hypoxic cells in spheroids and tumours. *Br. J. Cancer*, **56**, 395–400.

RALEIGH JA, LA DINE JK, CLINE JM AND THRALL DE. (1994). An enzyme-linked immunosorbent assay for hypoxia marker binding in tumours. *Br. J. Cancer*, **69**, 66–71.

ROJAS A, JOINER MC, HODGKISS RJ, CARL U, KJELLEN E AND WILSON GD. (1992). Enhancement of tumor radiosensitivity and reduced hypoxia-dependent binding of a 2-nitroimidazole with normobaric oxygen and carbogen: a therapeutic comparison with skin and kidneys. *Int. J. Radiat. Oncol. Biol. Phys.*, **23**, 361–366.

SMITHEN CE AND HARDY CR. (1982). The chemistry of nitroimidazole hypoxic cell radiosensitizers. In *The Chemistry of Nitroimidazole Hypoxic Cell Radiosensitizers*, Breccia A, Rimondi C and Adams GE. (eds) pp. 1–47. Plenum Press: New York.

THOMLINSON RH AND GRAY LH. (1955). The histological structure of some human lung cancers and the possible implications for radiotherapy. *Br. J. Cancer*, **9**, 539–549.

THRALL DE, MCENTEE MC, CLINE JM AND RALEIGH JA. (1994). ELISA quantification of CCI-103F binding in canine tumors prior to and during irradiation. *Int. J. Radiat. Oncol. Biol. Phys.*, **28**, 649–659.

THURMAN RG AND KAUFFMAN FC. (1985). Sublobular compartmentation of pharmacologic events (SCOPE): metabolic fluxes in periportal and pericentral regions of the liver lobule. *Hepatology*, **5**, 144–151.

THURMAN RG, JI S AND LEMASTERS JJ. (1986). Lobular oxygen gradients: possible role in alcohol-induced hepatotoxicity. In *Regulation of Hepatic Metabolism*, Thurman RG, Kauffman FC and Jungermann K (eds) pp. 293–320. Plenum Publishing Corporation: New York, NY.

VAN OS-CORBY DJ AND CHAPMAN JD. (1986). *In vitro* binding of $^{14}C$-misonidazole to hepatocytes and hepatoma cells. *Int. J. Radiat. Oncol. Biol. Phys.*, **12**, 1251–1254.

VAN OS-CORBY DJ, KOCH CJ AND CHAPMAN JD. (1987). Is misonidazole binding to mouse tissues a measure of cellular $pO_2$? *Biochem. Pharmacol.*, **36**, 3487–3494.

WATTS ME, DENNIS MF AND ROBERTS IJ. (1990). Radiosensitization by misonidazole, pimonidazole and azomycin and intracellular uptake in human tumor cell lines. *Int. J. Radiat. Biol.*, **57**, 361–372.

WOLPERT MF, ALTHAUS JR AND JOHNS DG. (1973). Nitroreductase activity of mammalian liver aldehyde oxidase. *J. Pharmacol. Exp. Ther.*, **185**, 202–213.